# Supplementary material for: Anti-Inflammatory and Anti-Bacterial Potential of Mulberry Leaf Extract on Oral Microorganisms
Source: Int J Environ Res Public Health. 2022 Apr 20;19(9):4984. doi: 10.3390/ijerph19094984 (PMC9099889; doi:10.3390/ijerph19094984)
Supplement: Supplementary file 1 [file ijerph-19-04984-s001.zip › ijerph-1651931-supplementary.pdf]

Anti-inflammatory and anti-bacterial potentials of mulberry leaf extract on oral microorganisms

Supplementary Materials and Methods

Cell culture

Immortalized human oral keratinocytes (IHOK) [1] and gingival fibroblast (hTERT-hNOFs) [2] were provided by the department of the Oral pathology in Yonsei University College of Dentistry. IHOK cells were immortalized by HPV16 E6/E7, and hTERT-hNOFs were immortalized by transfection with hTERT to be used *in vitro*. Both IHOK and hTERT-hNOFs cells were cultured in DMEM/F12 media (3:1 ratio) with 10% FBS and 1% penicillin/streptomycin at 37°C maintaining 5% CO_2 condition.

Cell viability test

IHOKs and hTERT-hNOFs were inoculated onto 96 well plate by $1x10^4$, and MTT (Duchefa) assay was performed after the treatment with each concentration of mulberry leaf extract for 24 hours.

Flow cytometry for ROS generation

To check the generation of ROS, IHOK (5×10^5 cells/well) and hTERT-hNOFs (4×10^5 cells/well) were seeded in the 6-well plate and pre-treated with the MAE by different concentrations (0.5, 1, and 2%) for an hour, and applied with 10 ng/ml LPS for 24 hours. Next, fluorescent probe 2′7′-dichlorofluorescin diacetate (H_2DCFDA) dye (Molecular Probes) was used. According to instruction, both cells were applied with 10 μM of H_2DCFDA dye in the dark at 37 °C for 20 min. ROS were analyzed by flow cytometry (Becton Dickinson, Beckman coulter).

Supplementary figures

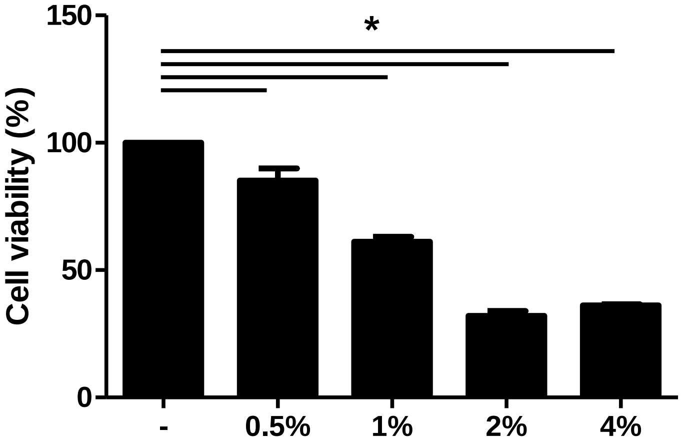

(a)

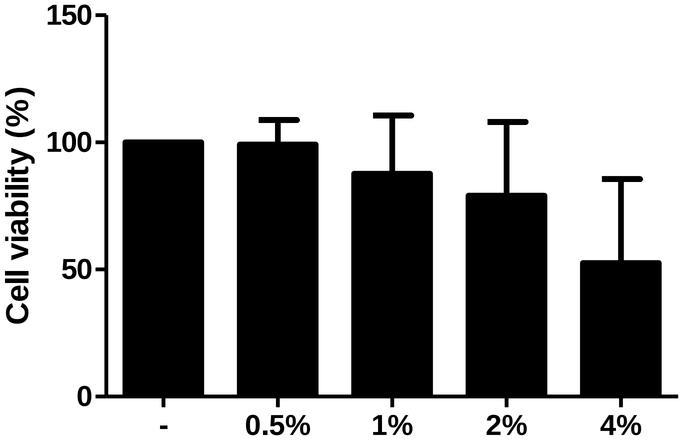

(b)

Figure S1. The cytotoxicity test by MAE in IHOKs and hTERT-hNOFs. IHOK (**a**) and hTERT-hNOFs (**b**) were seeded onto 96 well plates and were treated with the indicated concentrations (0.5,1,2 and 4%). The graphs were indicated to percentage of cell viability after normalization to control (untreated cells). The results are shown as mean value ± SD (n=3) (*P < 0.05).

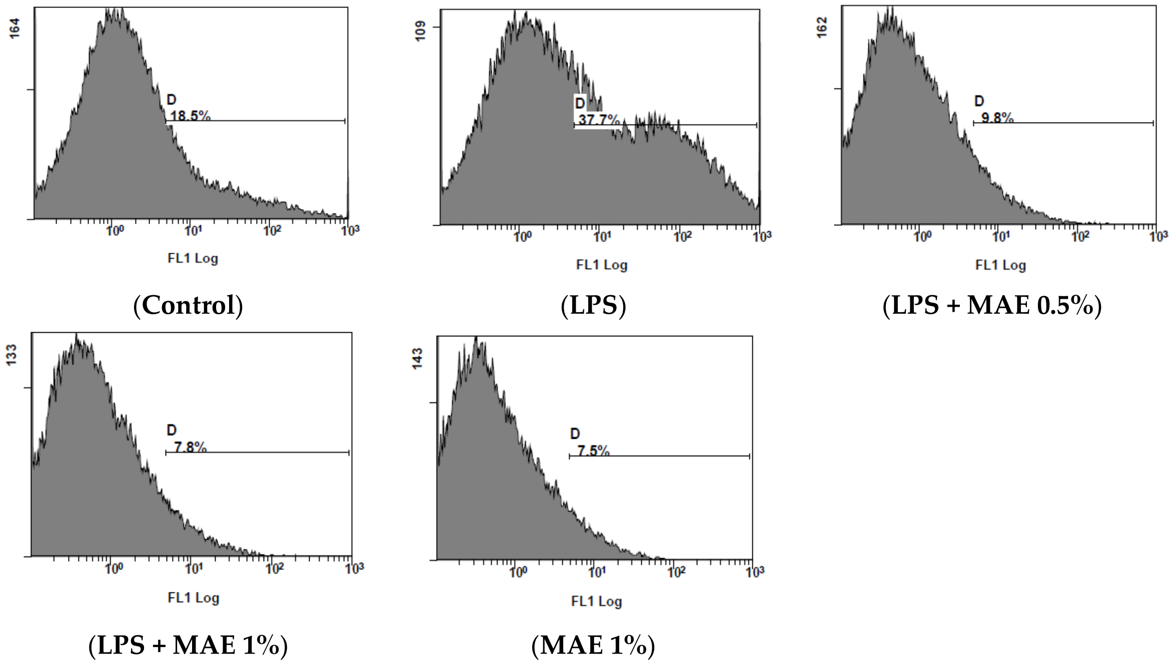

Figure S2. The ROS generation by MAE in IHOKs. IHOK cells (> 80% confluence of plates) were seeded onto 6 well plates and stabilized overnight. Next, cells were pre-applied with the concentration (0.5 and 1 %)of MAE and then treated LPS (10 ng/ml) for 24 hours. For measurement of ROS generation, fluorescent probe 2′7′-dichlorofluorescin diacetate (H_2DCFDA) dye was applied in each cell and then collecting cells were analyzed by flow cytometry. The histogram was shown to percentage of dye-positive cells.

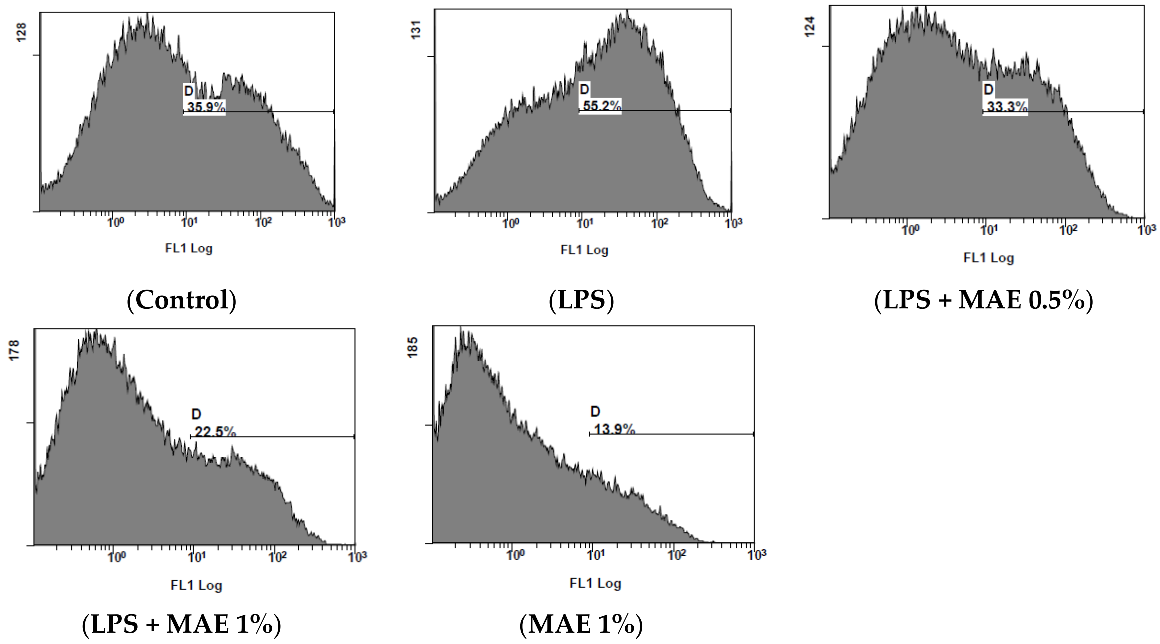

Figure S3. The ROS generation by MAE in hTERT-hNOFs. The hTERT-hNOFs (> 80% confluence of plates) were seeded onto 6 well plates and stabilized overnight. Next, cells were pre-applied with the concentration (1 and 2%) of MAE and then treated LPS (10 ng/ml) for 24 hours. For measurement of ROS generation, fluorescent probe 2′7′-dichlorofluorescin diacetate (H_2DCFDA) dye was applied in each cell and then collecting cells were analyzed by flow cytometry. The histogram was shown to percentage of dye-positive cells.

Supplementary references

1. Lee, H.J.; Guo, H.Y.; Lee, S.K.; Jeon, B.H.; Jun, C.D.; Lee, S.K.; Park, M.H.; Kim, E.C. Effects of nicotine on proliferation, cell cycle, and differentiation in immortalized and malignant oral keratinocytes. *J Oral Pathol Med* **2005**, *34*, 436-443.

2. Illeperuma, R.P.; Park, Y.J.; Kim, J.M.; Bae, J.Y.; Che, Z.M.; Son, H.K.; Han, M.R.; Kim, K.M.; Kim, J. Immortalized gingival fibroblasts as a cytotoxicity test model for dental materials. *J Mater Sci Mater Med* **2012**, *23*, 753-762.
